# Adrenergic tone benefits cardiac performance and warming tolerance in two teleost fishes that lack a coronary circulation

**DOI:** 10.1007/s00360-021-01359-9

**Published:** 2021-03-18

**Authors:** Andreas Ekström, Erika Sundell, Daniel Morgenroth, Erik Sandblom

**Affiliations:** Department of Biological and Environmental Sciences, University of Gothenburg, PO Box 463, 405 30 Gothenburg, Sweden

**Keywords:** Adrenergic, Atropine sulfate, Cholinergic, Critical thermal maximum, Heart rate

## Abstract

Tolerance to acute environmental warming in fish is partly governed by the functional capacity of the heart to increase systemic oxygen delivery at high temperatures. However, cardiac function typically deteriorates at high temperatures, due to declining heart rate and an impaired capacity to maintain or increase cardiac stroke volume, which in turn has been attributed to a deterioration of the electrical conductivity of cardiac tissues and/or an impaired cardiac oxygen supply. While autonomic regulation of the heart may benefit cardiac function during warming by improving myocardial oxygenation, contractility and conductivity, the role of these processes for determining whole animal thermal tolerance is not clear. This is in part because interpretations of previous pharmacological in vivo experiments in salmonids are ambiguous and were confounded by potential compensatory increases in coronary oxygen delivery to the myocardium. Here, we tested the previously advanced hypothesis that cardiac autonomic control benefits heart function and acute warming tolerance in perch (*Perca fluviatilis*) and roach (*Rutilus rutilus*); two species that lack coronary arteries and rely entirely on luminal venous oxygen supplies for cardiac oxygenation. Pharmacological blockade of β-adrenergic tone lowered the upper temperature where heart rate started to decline in both species, marking the onset of cardiac failure, and reduced the critical thermal maximum (CT_max_) in perch. Cholinergic (muscarinic) blockade had no effect on these thermal tolerance indices. Our findings are consistent with the hypothesis that adrenergic stimulation improves cardiac performance during acute warming, which, at least in perch, increases acute thermal tolerance.

**Supplementary Information:**

The online version contains supplementary material available at 10.1007/s00360-021-01359-9.

## Introduction

Upper thermal tolerance in fish (*i.e.*, the temperature beyond which the fish cannot survive) is partly governed by the cardiovascular system´s capacity to maintain tissue oxygen delivery during warming (see Eliason and Anttila 2017; Ekström et al. 2016a, 2019; Gollock et al. 2006; Badr et al. 2016). Acute warming typically results in elevated heart rate and cardiac output, but at temperatures approaching the upper thermal tolerance limit (here defined by the critical thermal maximum, CT_max_, in turn defined as the temperature at which the animal loses equilibrium (Beitinger et al. 2000)), a plateau and subsequent reduction in heart rate and cardiac output is often observed (Ekström et al. 2014; Ekström et al. 2016a; Heath and Hughes 1973; Hughes and Roberts 1970; Gollock et al. 2006). This decline in heart rate has been interpreted as cardiac failure in fish approaching CT_max_, and likely has multiple underlying reasons (see Eliason and Anttila 2017; Ekström et al. 2016a; Iftikar and Hickey 2013; Haverinen and Vornanen 2020; Vornanen 2020). First, the venous oxygen tension declines as temperature rises (*i.e.*, venous hypoxemia), which results in a reduced partial pressure gradient for oxygen diffusion into the spongy myocardium. The heart is an aerobic tissue and maintaining a sufficient cardiac oxygen supply is crucial for maintaining cardiac contractility at high temperatures (see Driedzic and Gesser 1994; Ekström et al. 2016a). Thus, exacerbated venous hypoxemia is particularly detrimental in fish species that lack a coronary arterial circulation where the luminal venous oxygen supply represents the sole route for myocardial oxygenation (Ekström et al. 2016a; Farrell and Smith 2017). Second, the increase in heart rate with rising temperatures may constrain luminal oxygenation further by reducing the time for oxygen diffusion as diastole shortens, along with increased myocardial diffusion distances as end-diastolic filling and distension decline (see Eliason and Anttila 2017). Finally, the specific tendency for heart rate to decline at high temperatures immediately prior to CT_max_ has recently been attributed to compromised ventricular myocardial excitability and action potential conduction capacity causing a functional atrioventricular (AV) block; *e.g*., as observed in roach (*Rutilus rutilus*), rainbow trout (*Oncorhynchus mykiss*) and brown trout (*Salmo trutta*)(Haverinen and Vornanen 2020; Badr et al. 2016; Haverinen et al. 2016; Vornanen 2020). The cellular mechanism behind this is an ensuing imbalance of Na^+^ and K^+^ ionic transmembrane flux rate dynamics due to deteriorating ion channel functionality (predominately Na^+^ channels) as temperature increases (Vornanen 2016, 2017, 2020).

The autonomic nervous system regulates cardiac activity in teleost fishes via stimulatory β-adrenergic and inhibitory cholinergic (muscarinic) pathways (Sandblom and Axelsson 2011; Nilsson 1983). Recently, adrenergic or cholinergic autonomic regulation of the heart was hypothesized to be important determinants of CT_max_ and overall cardiac performance during acute warming (Gilbert et al. 2019; Ekström et al. 2014, 2019). This stems from the idea that a controlled cholinergic slowing of heart rate at elevated temperatures could be beneficial by circumventing AV block by reducing the pacemaker rate to within the functional rate limits of the ventricle (Gilbert et al. 2019), lowering overall cardiac workload and oxygen demand, and/or improving myocardial oxygenation by extending the duration for luminal oxygen diffusion (Farrell 2007). Moreover, adrenergic stimulation via autonomic nerves or circulating catecholamines, the latter which increase with acute warming (Currie et al. 2013, 2008), could promote myocardial excitability and possibly also prevent AV block (Gilbert et al. 2019). Catecholamines are also well known to promote ventricular contractility in fish exposed to acute warming and hypoxia stress (Aho and Vornanen 2001; Hanson et al. 2006; Farrell et al. 1996). Several recent studies adressed these hypotheses experimentally, and tested whether pharmacological autonomic blockade alters CT_max_ and cardiac performance during warming using rainbow trout as model species (Gilbert et al. 2019; Ekström et al. 2014, 2019). Interestingly, while Gilbert et al. (2019) found that both cholinergic and β-adrenergic blockade with atropine and sotalol, respectively, reduced CT_max_ and the temperature at which heart rate started to decline in juvenile rainbow trout, we found no such effects when using a similar pharmacological protocol in adult trout (Ekström et al. 2014). Given the influence of the coronary circulation on cardiac perfromance during warming (Ekström et al. 2017), and that adult trout may be more dependent on this source of cardiac oxygenation as the amount of compact myocardium perfused by the coronaries increases with age (Farrell et al. 1988; Ekström et al. 2017; Brijs et al. 2016), we hypothesized that an increased coronary flow may have buffered any negative effects of the pharmacological blockade, which should have primarily impaired luminal oxygen diffusion. However, a follow-up study on adult rainbow trout with surgically ligated coronaries to exclude the potential influence of compensatory increases in coronary flow, similarly failed to replicate the earlier findings of Gilbert and co-workers, as thermal tolerance remained unaffected following subsequent pharmacological autonomic blockade (Ekström et al. 2019).

In the present study, we took a different approach to further explore the hypothesis that cardiac autonomic regulation enhances in vivo cardiac performance and CT_max_ in acutely warmed fish by examining two species; European perch (*Perca fluviatilis,* Linneaus 1758*)* and roach (*Rutilus rutilus*, Linneaus 1758), which both lack a coronary circulation and, thus, rely entirely on luminal venous oxygen supply for cardiac oxygenation. We specifically tested the prediction that pharmacological blockade of cholinergic or adrenergic cardiac control systems would reduce the temperature at which heart rate starts to decline during warming, and result in lowered CT_max_.

## Materials and methods

### Fish collection and holding

Perch were caught in the Baltic Sea close to Forsmark, Sweden (60°24′07.1"N 18°10′49.5"E) using hook and line during the late summer in 2018. They were transferred to a nearby laboratory facility (Sandblom et al. 2016a), and kept outdoors under natural light conditions in holding tanks (1200 L) receiving flow-through aerated brackish seawater (~ 5 ppt salinity) at ~ 17 °C. The fish were held for at least 3 days prior to experimentation and were not fed in captivity.

Roach were caught in baited traps in lake Rådasjön near Gothenburg, Sweden (57°39′30.8"N 12°04′35.5"E) during late autumn in 2016 and 2017. They were transferred to holding tanks (1200 L) receiving recirculating aerated freshwater in the animal facility at the University of Gothenburg, and were acclimated to 10 °C and a 12:12-h light:dark photoperiod for at least 4 weeks prior to the experiments. Roach were fed commercial fish pellets 3 times per week, but feeding was stopped one week before experimentation. The acclimation temperatures in the holding tanks for both species reflected the temperatures at the collections sites at the time of capture. All procedures were approved by ethical permit #165–2015 issued by the regional animal ethics committee.

### Anesthesia and surgery

Perch and roach were anesthetized in ~ 18 °C seawater (5 ppt) and 10 °C freshwater, respectively, containing MS-222 (Tricaine methanesulfonate; 100 mg L^−1^ and 150 mg L^−1^, respectively) buffered with NaHCO_3_ (300 mg L^−1^, roach only). Body mass and length were determined and the fish were placed ventral side up on wet foam on a surgery table. Surgical anesthesia was maintained by continuously irrigating the gills with 10 °C water containing a lower dose of MS-222 (50–75 mg L^−1^) buffered with NaHCO_3_ (150 mg L^−1^, roach only). To record heart rate, two ECG electrodes (AS 631–2, Cooner wire, Chatsworth, CA) were inserted subcutaneously between the pectoral fins in a ventral medial position using a 23 gauge needle. The electrodes were inserted at a ~ 45° angle placing the electrode tips on either side of the heart. For administration of pharmacological substances, a PE-50 catheter was inserted into the abdominal cavity, 3–4 cm posterior to the pectoral fin (Ekström et al. 2016b). The wire and catheter were secured to the skin using 4–0 silk sutures. The fish were then placed in opaque experimental chambers (width: 130 mm; length: 340 mm; height: 170 mm), which received a continuous flow of aerated water identical to the respective holding tanks, and were allowed to recovered for at least 24 h before experiments started.

### Experimental protocol

Resting heart rate was first recorded for at least 2 h in perch and roach at 18 or 10 °C, respectively. A bolus injection (1 ml kg^−1^) of either saline (0.9% NaCl, Control), atropine sulfate (1.2 mg kg^−1^), sotalol (2,7 mg kg^−1^) or propranolol (3 mg kg^−1^, roach only) was then administered via the abdominal catheter in separate groups of fish. A 0.5 ml bolus of saline was injected to flush the catheter dead space. When stable post-injection heart rate values had been attained at the respective acclimation temperature (typically within 30–60 min), fish were subjected to an acute thermal challenge. In the perch, the temperature was first raised from 18 °C to 23 °C in 1 h (*i.e.*, 5 °C h^−1^), followed by a heating rate of 3 °C h^−1^ until the fish lost equilibrium, *i.e.*, CT_max_. In roach, the temperature was raised from 10 °C to 20 °C in 2 h (*i.e.*, 5 °C h^−1^), followed by 3 °C h^−1^ until CT_max_. All pharmacological substances and chemicals were purchased from Sigma-Aldrich (St Louis, MO, USA).

### Data acquisition and calculations

The ECG electrodes were connected to bioamplifiers (ML136, AD instruments, Castle Hill, Australia; Range: 10 mV; Low-pass filter: 1 kHz; High-pass filter: 0.3 Hz; 50 Hz notch filter). The water temperature was recorded continuously using a custom-built temperature logger (EW 7221, Crn Tecnopart, Barcelona, Spain). Analog outputs from the recording equipment were relayed to a PowerLab system (AD Instruments, Sydney, Australia) connected to a computer running Labchart Pro software (v7.2.2, AD Instruments, Castle Hill, Australia).

The raw ECG signals were filtered and optimized (Band-pass digital filters in Labchart; High cut-off frequency range: 15–40 Hz; Low-pass range: 1–4 Hz) and heart rate was determined from the rate of ventricular depolarizations (*i.e.*, R peaks) in the ECG. For each fish, we determined the highest heart rate during warming where heart rate peaked or plateaued (*i.e.*, peak heart rate), which was typically followed by a progressive decline due to cardiac arrhythmias and/or a prolonged inter-beat interval. We also determined the temperature at which peak heart rate occurred. Fish condition factor was calculated as:

$$\quad {\text{Condition factor}} = (100 \times {\text{Body mass}})/{\text{Body length}}^{3}$$

using body mass in grams and length in centimeters.

### Statistics

Statistical analyses were performed using SPSS (v. 25, SPSS Inc., Chicago, IL, USA). Values are presented as means ± S.E.M unless otherwise stated. Normality and homogeneity of variances were determined using Shapiro–Wilk’s and Levene’s tests, respectively. One-way ANOVA´s or Kruskal–Wallis H-tests were performed to compare pre-treatment routine heart rate, peak heart rate, temperature for the peak heart rate, CT_max_, body mass, length and condition factor among treatment groups. Paired t-tests were used to compare pre-, and post-injection heart rate within groups. Linear mixed models were used to evaluate the effects of temperature on heart rate (repeated measures) within treatment groups and for among-treatment comparisons across temperatures. Temperature, treatment and their interaction (*i.e.*, temperature*treatment) were modeled. A first-order autoregressive covariance structure provided the best fit to the data, as indicated by the lowest Akaike´s information criterion (AIC). Only data points until the lowest temperature at which peak heart rate occurred in an individual fish across treatment groups (*i.e.*, at 27 and 24 °C for perch and roach, respectively) were included in this analysis. Statistically significant main effects were further explored by pair-wise comparisons among experimental groups. Statistical significance was accepted at *p* ≤ 0.05.

## Results

Body mass, length and condition factor did not differ among groups of perch (Table 1). However, sotalol-, and propranolol-treated roach (caught 2017) had lower body mass (~ 43%, *F*_3_ = 15.0, *p* < 0.001), length (~ 15%, *F*_3_ = 13.2, *p* < 0.001) and condition factor (~ 10%, *F*_3_ = 4.4, *p* = 0.019, sotalol only) compared to controls (caught in 2016).

**Table 1:** Morphological characteristics for the experimental groups of European perch (*Perca fluviatilis*) and roach (*Rutilus rutilus*)

	European perch			Roach			
	Control	Atropine	Sotalol	Control	Atropine	Sotalol	Propranolol
Body mass (g)	123.9 ± 8.7	125.4 ± 9.1	127.9 ± 8.4	76.8 ± 4.8^a^	74.3 ± 5.9^a^	42.8 ± 2.5^b^	42.9 ± 5.9^b^
Body length (mm)	198.8 ± 5.0	200.6 ± 4.7	199.5 ± 3.7	167.2 ± 3.7^a^	165.5 ± 3.9^a^	140.7 ± 2.5^b^	142.2 ± 5.8^b^
Condition factor	1.55 ± 0.04	1.52 ± 0.03	1.58 ± 0.04	1.63 ± 0.05^a^	1.62 ± 0.06^a^	1.51 ± 0.03^a^	1.43 ± 0.04^b^

Dissimilar letters denote statistically significant (*p* < 0.05) differences among groups

**Fig. 1:**
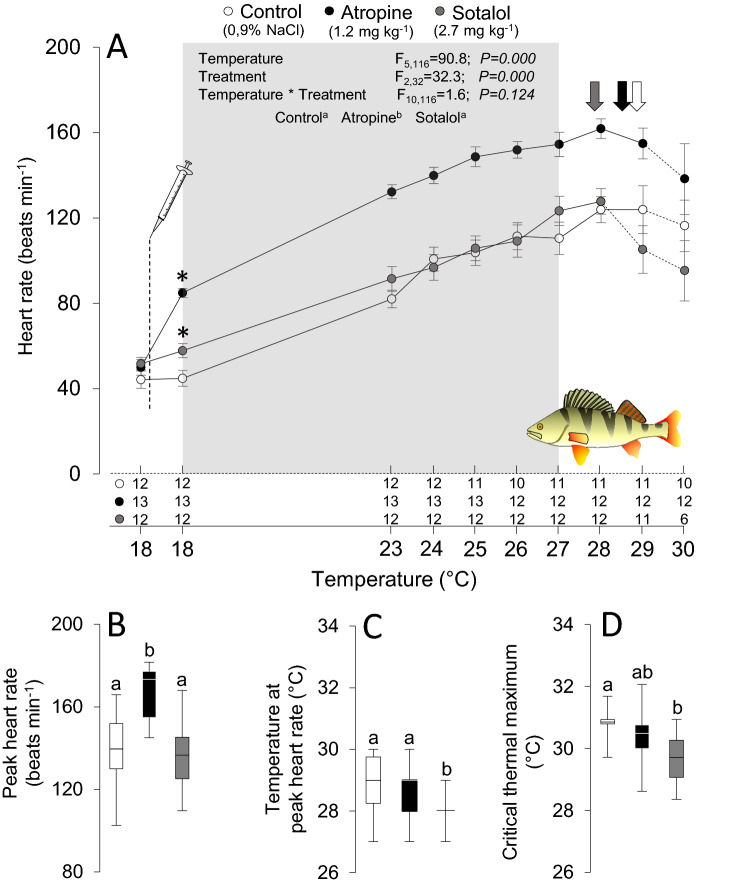
Effects of acute warming on heart rate and the critical thermal maximum in European perch, *Perca fluviatilis*. Heart rate (**a**), peak heart rate (**b**), temperature for the peak heart rate (**c**) and the critical thermal maximum (**d**) in perch pharmacologically treated with saline (0.9% NaCl) as control (white), atropine (black) or sotalol (gray). The sample sizes (numbers above X-axis) during the thermal ramping in panel A changed from the start to the end of the heating protocol among groups, either as the ECG signal was lost or individual fish reached their critical thermal maximum. *Denotes statistically significant (*p* ≤ 0.05) effects on heart rate within groups following the pharmacological treatments. The inset table shows the statistical details from the mixed model analyzing the effects of autonomic blockade on heart rate between 18 and 27 °C, the latter being the lowest temperature at which heart rate started to decline in a single individual fish across treatment groups (indicated by shaded area). The color-coded vertical arrows indicate the average temperature at which peak heart rate occurred in each treatment group, and data points beyond peak heart rate are indicated by dashed connecting lines. Dissimilar letters denote statistically significant treatment effects among experimental groups (color figure online)

**Fig. 2:**
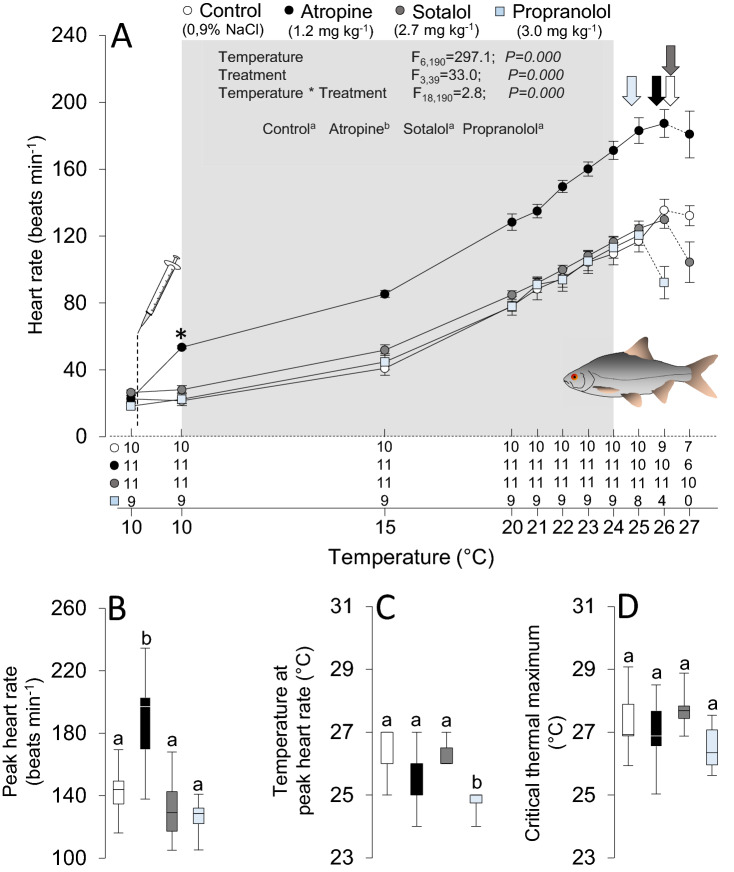
Effects of acute warming on heart rate and the critical thermal maximum in common roach, *Rutilus rutilus*. Heart rate (**a**), peak heart rate (**b**), temperature for the peak heart rate (**c**) and the critical thermal maximum (**d**) in roach pharmacologically treated with either saline (0.9% NaCl) (Control, white), atropine (black), sotalol (gray) or propranolol (light blue). The sample sizes (numbers above X-axis) during the thermal ramping in panel A changed from the start to the end of the heating protocol among groups, either as the ECG signal was lost or individual fish reached their critical thermal maximum. *Denotes statistically significant (*p* ≤ 0.05) on heart rate within groups following the pharmacological treatments. The inset table depicts the statistical details from the mixed model analyzing the effects of autonomic blockade on heart rate between 10 and 24 °C, the latter being the lowest temperature at which heart rate started to decline in a single individual fish across treatment groups (indicated by shaded area). The color-coded vertical arrows indicate the average temperature at which peak heart rate occurred in each treatment group, and data points beyond peak heart rate are indicated by dashed connecting lines. Dissimilar letters denote statistically significant treatment effects among experimental groups (color figure online)

Routine heart rate significantly increased in atropine-treated perch at 18 °C and roach at 10 °C by 35 beats min^−1^ (41%) and 31 beats min^−1^ (57%), respectively (Figs. 1a and 2a). However, β-adrenergic blockade with sotalol did not significantly alter routine heart rate in either species (Figs. 1a and 2a). Due to the lack of effect of sotalol, we also treated roach with another β-blocker, propranolol, which similarly did not affect routine heart rate at 10 °C (Fig. 2a).

Acute warming elevated heart rate across species and treatment groups, but the heart rate of atropine-treated fish always remained significantly elevated compared to the control groups in both species throughout the acute warming protocol (Figs. 1a and 2a). In perch, this meant that peak heart rate was higher in atropinized fish relative to controls (166 *vs*. 138 beats min^−1^; *F*_2_ = 8.9, *p* = 0.001, Fig. 1b), but there was no difference in temperature for the peak heart rate (28.6 ± 0.3 *vs*. 28.9 ± 0.3 °C, Fig. 1c) or at the CT_max_ (30.4 ± 0.2 *vs*. 30.8 ± 0.2 °C, Fig. 1d). While peak heart rate in perch after β-adrenergic blockade with sotalol was similar (137 beats min^−1^) to the control group, the β-adrenergic blockade significantly reduced both the temperature for the peak heart rate (by 1.0 °C to 27.9 ± 0.2 °C*; F*_2_ = 8.9, *p* = 0.041) and CT_max_ (by 1.1 °C to 29.7 ± 0.2 °C*; F*_2_ = 6.5, *p* = 0.003).

Similarly, in roach, peak heart rate was also higher after atropine relative to control fish (190 *vs*. 143 beats min^−1^; *F*_3_ = 17.7, *p* < 0.001, Fig. 2b), but there were no differences in the temperature for the peak heart rate (25.7 ± 0.3 *vs*. 26.3 ± 0.3 °C, Fig. 2c) or CT_max_ (27.0 ± 0.3 *vs.* 27.3 ± 0.3 °C¸ Fig. 2d). Moreover, peak heart rate in roach was also unaffected by both sotalol and propranolol (132 and 126 beats min^−1^, respectively), relative to the control group. Yet, heart rate peaked at a lower temperature in roach treated with propranolol (24.8 ± 0.2 °C; *F*_3_ = 10.5, *p* < 0.001, Fig. 2c). There were no differences in the temperature for the peak heart rate between controls and sotalol-treated roach (26.3 ± 0.1 °C), and CT_max_ was unaffected by both sotalol and propranolol treatment in roach (27.7 ± 0.2 and 26.5 ± 0.2 °C, respectively, Fig. 2d). It is also worth noting that there was a close to significant trend towards a lower CT_max_ in propranolol-, *vs*. sotatol-treated fish (by 1.2 °C, *p* = 0.051). Peak heart rate always occurred at lower temperatures than CT_max_ in individual fish across species and treatment groups (Figs. 1c, d; 2c, d).

In roughly 50–60% of individual experiments, the in vivo ECG traces at the highest temperatures were of sufficient quality to allow more detailed analyses (*e.g.,* individual P waves and/or QRS complexes were clearly distinguishable). In both species, the decline in heart rate immediately prior to CT_max_ (see Figs. 1a and 2a) was either associated with an arrhythmic heartbeat as indicated by irregular heart rhythm and/or occasional missing QRS complexes, or a gradual decline in ventricular depolarization rate without arrhythmias. Although the quality of the ECG signal prevented us from assessing the presence of arrhythmias in all fish, a general pattern emerged where adrenergically blocked fish of both species appeared to display a higher prevalence of arrhythmias at high temperatures relative to controls (Figs. 1a and 2a, Table 2). In contrast, none of the atropine-treated perch and roach displayed any obvious signs of arrhythmias.

**Table 2:** Prevalence of arrhythmia during acute warming in European perch (*Perca fluviatilis*) and roach (*Rutilus rutilus*)

	European perch			Roach			
	Control	Atropine	Sotalol	Control	Atropine	Sotalol	Propranolol
Sample size (*n*)	8	9	6	8	7	11	6
Arrhythmia prevalence (%)	50	0	100	63	0	73	83

## Discussion

In accordance with our hypothesis, adrenergic blockade impaired routine cardiac function at higher temperatures as indicated by the reduced temperature at which heart rate peaked in both species. This suggests that adrenergic stimulation has a beneficial and potentially protective influence on in vivo heart function during acute warming, which was also shown by Gilbert and co-workers in rainbow trout (2019). Moreover, as suggested by these authors (2019), adrenergic stimulation may rectify the imbalances in ion (Na^+^ and K^+^) flux rate dynamics which underlie the failure of myocardial action potential conduction and cardiac excitability, thus preventing AV block and/or arrhythmias which are associated with the declining heart rate at high temperatures (Aho and Vornanen 2001; Vornanen 2017; Haverinen and Vornanen 2020). Indeed, the higher prevalence of arrhythmias after β-adrenergic blockade in both species observed here may reflect an accentuation of AV block at higher temperatures, which warrants further exploration.

In perch, the reduced temperature for the peak heart rate following β-adrenergic blockade was associated with a lower CT_max_. This may have reflected that the lack of adrenergic tone on the heart led to greater impairments of stroke volume and cardiac output during warming, compromising oxygen and nutrient delivery to essential tissues such as the brain. Indeed, β-adrenergic stimulation is known to have a protective impact on cardiac contractility during adverse extracellular conditions exaggerated by acute warming, *e.g*., hypoxia, acidosis or hyperkalemia (Hanson et al. 2006; Farrell et al. 1996; Roberts and Syme 2018). In contrast, however, none of the adrenergic antagonists affected CT_max_ in roach, although the β-adrenergic blockade with propranolol reduced the temperature at which heart rate peaked. It is possible that roach had a better capacity to maintain cardiac output and tissue oxygen delivery through compensatory increases in stroke volume at temperatures above the temperature at which heart rate peaked. Another possibility is that the central nervous system in roach is better able to withstand a brief period of insufficient blood perfusion such that overall thermal tolerance was unaffected. However, it is also important to emphasize that CT_max_ is not only governed by cardiovascular oxygen delivery failure, but has also been linked to failing mitochondrial and/or neural functions in fish and other aquatic ectotherms at extreme high temperatures (MacMillan 2019; O'Brien et al. 2018; Friedlander et al. 1976; Iftikar and Hickey 2013; Vornanen 2020). The fact that the two β-adrenergic antagonists affected temperature for the peak heart rate differently in roach is enigmatic, but may relate to differences in the antagonizing effects elicited by these agents on cardiac function. For example, in cats, propranolol induced larger reductions in stroke volume and cardiac output relative to treatment with sotalol (Åberg et al. 1969). Further, we cannot exclude the possibility that the pharmacological β-adrenergic blockade induced off-target effects to different extents, which may have affected the responses observed here, *e.g*., by affecting vascular resistance and blood pressure (Sandblom and Axelsson 2011), altering the oxygen carrying capacity of the blood (Perry and Bernier 1999; Nikinmaa 1992), and/or by blocking voltage gated cardiac and brain Na^+^ channels as observed in vitro in human cells (Wang et al. 2010). Our findings highlight that a detailed assessment of potential differences in the inhibitory potency of these β-adrenergic antagonists on cardiac function in different fish species is warranted.

Contrasting with our hypothesis, atropine did not affect CT_max_ or the temperature at which heart rate peaked in either species. This is consistent with our previous observation in adult rainbow trout (Ekström et al. 2014, 2019), but contrasts with observations in juvenile trout where atropine reduced CT_max_ and the temperature at which heart rate peaked (Gilbert et al. 2019). Gilbert and colleagues (2019) speculated that cholinergic slowing of action potential generation in the cardiac pacemaker may serve to synchronize the pacemaker rate with the functional depolarisation rate of the ventricle, thus avoiding the AV block that may occur at higher temperatures (Haverinen and Vornanen 2020). However, we found no indications (0% occurrence) of AV block following cholinergic blockade in either species. It is possible that the abolishment of the presumed positive effects of cholinergic slowing of heart rate was compensated for by an increased adrenergic tone on the heart of both perch and roach, which may have augmented ventricular excitability, contractility and overall cardiac function as discussed above.

In contrast to previous studies in perch (Sandblom et al. 2016b; Sandblom and Axelsson 2011), as well as numerous other teleosts (Ekström et al. 2014, 2019; Gilbert et al. 2019; Vornanen 2017; Altimiras et al. 1997), β-adrenergic blockade did not affect routine heart rate in perch or roach in the present study. This could reflect a low adrenergic tone in these species during the resting conditions and at the temperatures evaluated here. Indeed, perch from this area have previously been shown to maintain a relatively low (~ 10%) adrenergic tone on the heart at rest at similar acclimation temperatures (Sandblom et al. 2016a, b). We are, however, unaware of previous measurements of adrenergic tone in roach. The unaltered heart rate following the sotalol and propranolol treatments could also reflect a compensatory release of cholinergic tone after the β-adrenergic blockade, which kept heart rate unchanged. Consistent with the previous observations in various teleosts (see Axelsson et al. 2000; Ekström et al. 2019; Ekström et al. 2014; Gilbert et al. 2019; Sandblom et al. 2016b; see Vornanen 2017), both perch and roach exhibited relatively high resting cholinergic tones at their respective acclimation temperatures, as well as during acute warming. This was manifested by considerable elevations in heart rate across temperatures following atropine treatment (by 41 and 57%, respectively). We have previously shown that the cholinergic tone may vary from ~ 30 to 70% in perch from this region at similar temperatures (Sandblom et al. 2016a, b), but again, we are unaware of any previous reports of autonomic tones in roach.

It is largely unknown whether and to what extent the recent thermal history affects the influence of autonomic regulation on cardiac performance and whole animal tolerance during acute warming in fish as explored here. What is known, however, is that thermal acclimation may lead to substantial changes in basal autonomic tones in fish (Sandblom et al. 2016a, b; Ekström et al. 2016b; Wood et al. 1979), which could possibly affect the scope for autonomic regulation during warming. For example, elevations in both cholinergic and adrenergic tones were observed when comparing a population of chronically warmed European perch (~ 22–25 °C) to a population from a cooler reference habitat (16–19 °C) (Sandblom et al. 2016a, b). Furthermore, while chronic warming elevates cholinergic but not adrenergic tone in rainbow trout (Ekström et al. 2016b; Wood et al. 1979), cold acclimation may reduce the adrenergic sensitivity in this species (Keen et al. 1993). Assessing how shifting thermal acclimation regimes affect autonomic influence on cardiac performance during acute warming or CT_max_ remains an interesting venue for future exploration.

In summary, our results show that blockade of β-adrenergic, but not cholinergic, cardiac autonomic control systems has a negative influence on cardiac performance during warming in perch and roach; two species that lack coronaries and solely rely on luminal oxygen supply to the heart. Moreover, adrenergic stimulation of the heart appears to be associated with improved acute warming tolerance in perch. Thus far, the current and previous findings (Ekström et al. 2014, 2019; Gilbert et al. 2019) have not provided a uniform picture of the extent to which autonomic cardiac regulation augments cardiac performance and upper thermal tolerance limits in fish. While these discrepancies in experimental outcomes of the current and prior studies might be explained by the slight differences in methodological approaches employed across studies to test these hypotheses, it also likely reflects different inter-, and intra-species specific physiological responses and capacities in coping with a warming environment.

## Supplementary Information

Below is the link to the electronic supplementary material.

Electronic supplementary material 1 (XLSX 39 kb)

## Data Availability

The dataset from which the results of the current study are based is available as online supplementary information.
